# Spinning plates: livelihood mobility, household responsibility and anti‐retroviral treatment in an urban Zambian community during the HPTN 071 (PopART) study

**DOI:** 10.1002/jia2.25117

**Published:** 2018-07-19

**Authors:** Virginia Bond, Fredrick Ngwenya, Angelique Thomas, Melvin Simuyaba, Graeme Hoddinott, Sarah Fidler, Richard Hayes, Helen Ayles, Janet Seeley, the HPTN 071 (PopART) study team

**Affiliations:** ^1^ Zambart, School of Medicine University of Zambia Lusaka Zambia; ^2^ Department of Global Health and Development Faculty of Public Health and Policy London School of Hygiene and Tropical Medicine London UK; ^3^ Desmond Tutu TB Centre Stellenbosch University Tygerberg South Africa; ^4^ Department of Medicine Imperial College London St Mary's Campus London UK; ^5^ Department of Infectious Disease Epidemiology London School of Hygiene and Tropical Medicine London UK; ^6^ Department of Clinical Research London School of Hygiene and Tropical Medicine London UK; ^7^ MRC/UVRI Uganda Research Unit on AIDS Entebbe Uganda

**Keywords:** Zambia, Livelihood Mobility, HIV, ART, Time‐Geography, Household responsibility

## Abstract

**Introduction:**

Qualitative data are lacking on the impact of mobility among people living with HIV (PLHIV) and their decision‐making around anti‐retroviral treatment (ART). We describe challenges of juggling household responsibility, livelihood mobility and HIV management for six PLHIV in urban Zambia.

**Methods:**

Six PLHIV (three men and three women, aged 21 to 44) were recruited from different geographic zones in one urban community drawn from a qualitative cohort in a social science component of a cluster‐randomized trial (HPTN071 PopART). Participants were on ART (n = 2), not on ART (n = 2) and had started and stopped ART (n = 2). At least two in‐depth interviews and participant observations, and three drop‐in household visits with each were carried out between February and August 2017. Themed and comparative analysis was conducted.

**Results:**

The six participants relied on the informal economy to meet basic household needs. Routine livelihood mobility, either within the community and to a nearby town centre, or further afield for longer periods of time, was essential to get by. Although aware of ART benefits, only one of the six participants managed to successfully access and sustain treatment. The other five struggled to find time to access ART alongside other priorities, routine mobility and when daily routines were more chaotic. Difficulty in accessing ART was exacerbated by local health facility factors (congestion, a culture of reprimanding PLHIV who miss appointments, sporadic rationed drug supply), stigma and more limited social capital.

**Conclusions:**

Using a time‐space framework illustrated how household responsibility, livelihood mobility and HIV management every day were like spinning plates, each liable to topple and demanding constant attention. If universal lifelong ART is to be delivered, the current service model needs to adjust the limited time that some PLHIV have to access ART because of household responsibilities and the need to earn a living moving around, often away from home. Practical strategies that could facilitate ART access in the context of livelihood mobility include challenging the practice of reprimand, improving drug supply, having ART services more widely distributed, mapped and available at night and weekends, and an effective centralized client health information system.

## 1 Introduction

In 2016, Zambia had an estimated 59,000 new HIV infections and approximately 21,000 AIDS‐related deaths. There were 1,200,000 people living with HIV (PLHIV) among whom 65% had access to antiretroviral therapy (ART) and an estimated 58% had attained viral suppression 1. Reaching universality of testing and treatment for HIV in all populations in Zambia requires addressing patterns of mobility including those common to the day‐to‐day experiences of PLHIV. Data in sub‐Saharan Africa show mobility as a risk factor for acquiring HIV 2 and a reason for not engaging with care 3. In this paper, we use qualitative data to illustrate how the convergence of livelihood mobility, household responsibility and HIV creates time and space challenges for ART access that were, or could be, reduced by adjustments made both by individual PLHIV and the health system.

The most prevalent form of mobility in Zambia is linked directly to livelihoods. In the absence of a comprehensive welfare system, economic survival is often dependent on the ability to trade in assorted goods, casual work in construction and mines, charcoal making and more seasonal trading in fish and maize. Mobility patterns linked to these economic activities involve four types of movement: rural‐rural, rural‐urban, urban‐rural and urban‐urban, labelled by Bruijn et al. “the rural‐urban dichotomy” 4. For PLHIV who are dependent on mobile livelihoods, immediate economic survival can come at a cost to their long‐term health needs 5. When PLHIV weigh up the relative costs and benefits of various courses of action in their daily life and choose the course that provides “the best return” [6 p1469], earning money can be prioritized over frequent clinic appointments 7, 8. The need to prioritize livelihood needs intensifies with deepening social obligations, particularly for the head of the household and in the wake of deaths or lower productivity due to HIV 9.

Individuals living with HIV with frequent livelihood commitments and many social‐network orientated responsibilities have “compressed temporal and spatial windows” [9 p853]. Harvey's concept of “time‐space compression” 10, which refers to any alteration in the quality and relationship between space and time, helps convey how ART constrains PLHIV pursuing economic activities. PLHIV, for example, are often expected to collect ART on a routine basis from a specific HIV service close to their main residence but their household livelihood needs may require time, more movement and flexibility 11, 12, 13, 14, 15, 16.

Takahashi et al. 9 proposed a time‐space typology of rigid, flexible and chaotic daily routines to illustrate the challenge of fitting ART obligations alongside multiple obligations [9 p861]. A systematic review by Mills et al. 17 on patient reported barriers to adherence, identified disruptions in routine or having a chaotic schedule, and difficulties coordinating adherence with work, family, or caregiving responsibilities as barriers to adherence. There is no smooth, static “fit” between the competing needs of raising money and managing HIV successfully, and poverty and temporal and spatial compression exacerbate the tensions between the two 9, 10, 11, 16, 18, 19, 20.

Quantitative data in sub‐Saharan Africa have shown mobility as a risk factor both for acquiring HIV 2 and a reason for not engaging or disengaging from care 3. Few qualitative data exist on access to care and treatment to provide a deeper understanding of how PLHIV and their households navigate different priorities 9, 21, 22.

Focusing on an urban community of 37,034 people (18,517 aged 18 and over) and 8484 households with a HIV prevalence of 14% 23, we use six case‐studies of adult residents living with HIV (LHIV) to illustrate the dilemmas posed by livelihood‐related mobility and the obligations of anti‐retroviral treatment (ART). Drawing on the considerations of time and space that deliberate the impact of geographic mobility on time to access HIV health services 9, 24, we illustrate PLHIV juggling to balance livelihood mobility, household responsibilities and their health, using an analogy of spinning plates.

## 2 Methods

Data are drawn from the social science component of HIV Prevention Trials Network (HPTN) 071 (Population Effects of Antiretroviral Therapy to Reduce HIV Transmission, PopART) study, a cluster randomized trial (HPTN071) measuring the impact of a combination HIV prevention package (including Universal HIV Testing and Treatment [UTT]) on population level HIV incidence 2014 to 2017 25. For the intervention, a cadre of local community lay workers employed by the trial provided a broad package of HIV services at household level, referring clients to the local government health facility for ART 26. The HPTN071 (PopART) social science component runs from the start of the trial to the end of follow up (2012 to 2018). This includes a qualitative cohort (2016 to 2017) using participatory and ethnographic approaches to explore responses to HIV testing and treatment over time and across different social groups, contexts and HIV outcomes in the final year of the intervention. The qualitative cohort data collection with adults was carried out in four Zambian HPTN071 (PopART) communities selected for representing different intervention packages and geographical areas. A total of 39 adult participants (19 men and 20 women) were purposefully selected to represent different HIV pathways, geographical locations and gender and age groups. Twenty‐one were LHIV (out of whom nine were on treatment, eight had defaulted and four had not started treatment). No participant was selected based on mobility type.

This analysis focuses on qualitative cohort data from six of these participants in one of these four Zambian communities. The community (referred to as “Zx” in this manuscript) was selected because the universal treatment intervention was in place and for demonstrating (due to location and a dominant lower socio‐economic class) salient livelihood mobility patterns that are typical of the Zambian informal economy. We focus on the six adult participants LHIV (three men, three women – aged 21 to 44 years) who, at recruitment, had either not started ART (n = 2), were on ART (n = 2) or had started and stopped ART (n = 2). The purpose of selecting these six adult participants is to provide illustrative and detailed case material 27 which address the influences of livelihood and mobility and household responsibilities on decisions to start and stay on ART. The competing priorities of providing for the household and managing HIV was a broader theme apparent in the communities 28. Adopting a case study approach allows the complexity of livelihoods, mobility and health systems to be conveyed in detail, while acknowledging the uniqueness and particularity of each case 29.

In February 2017, the six participants were recruited from different geographic zones in Zx using the knowledge and introductions of community lay workers. Data were collected by a Zambian social scientist supported by a local research assistant using in‐depth interviews (IDIs) and participant observation during visits every three months, with additional monthly drop‐in visits to maintain contact. By August 2017, at least two IDIs with two observations and three research assistant drop‐in visits had been carried out with each participant and their household. Interview discussion guides focussed on questions around family and kinship; understandings of mobility, place and space; HIV and household livelihood; HIV, gender relations and stigma; and lastly, sex, love, marriage and HIV. Interviews took three hours on average and were usually carried out around daily tasks of participants, taking breaks when necessary or coming back another day to finish. The mobility of participants and the absence of incentives extended time in the field, with repeated visits often necessary. Observations were carried out in the households at the same time as both interviews and other visits, and carried out for one day at the local health facility, observing client flow from early in the morning to the afternoon. Drop‐in visits by research assistants furnished details on the whereabouts, health and details of the household of the participant using a structured check list. IDIs were recorded with participants’ consent, and handwritten notes were taken. Field notes and sketches were made during observations.

Social science team debriefings after each block of fieldwork aided discussions around emerging themes. Notes and summaries of each interview were written up and IDIs transcribed by the researchers. Comparative, themed analysis was carried out inductively and manually on the raw data by the first authors. Once the juggling between livelihood mobility, household responsibility and HIV emerged, all the data supporting these themes was identified and collated, and analysed for each individual case‐study and across the six case‐studies. The analysis was led by the two first authors and findings were also discussed with other social scientists who carried out the qualitative cohort fieldwork in the other three sites.

### 2.1 Ethical approvals

The Biomedical Research Ethics Committee of University of Zambia, the London School of Hygiene and Tropical Medicine Ethics Committee and the Zambian government approved this research. Participants were asked to participate on a voluntary basis without receiving any incentives, and written informed consent was obtained before the start of the first research activity. Staff members in charge of the health facility were asked for permission before carrying out the health facility observation. Informed consent and interviews were conducted in local languages chosen by participants. The community name is anonymized and pseudonyms used for participants in this manuscript to protect participant identity.

## 3 Results

### 3.1 Intervention context

Data from the trial showed high rates of mobility in the eight intervention Zambian communities 23, 28, 30, 31. This mobility was both within communities and in and out of communities, and made it hard to reach mobile individuals for testing (especially men) and undermined uptake of treatment in more communities where mobility was more prominent 31, 32, 33, and linkage of mobile PLHIV into care. Zx is a densely populated unplanned settlement on the outskirts of a district town [Bond, Simuyaba et al. Narrative Report 2013, internal technical report, Zambart, Lusaka]. Livelihood mobility in Zx included residents moving for trading and other casual work within the community (3 to 15 km), moving into a nearby town centre (5 to 20 km) and/or fishing camp (90 km) either using public mini‐buses, bicycles or by foot, and travelling some distance (usually 200 to 500 km) to other provincial centres, rural areas and markets using buses and lorries.

The one government health facility within Zx had an ART clinic. Two other options for accessing ART in or near Zx were a Non‐Governmental Organization (NGO) centre within and a renovated government health facility just outside the community boundary. The NGO centre was a small health facility about 12 km from the local government health facility which provided daily HIV services (testing and ART) with the mandate and supplies of the government. Further afield there were other options, including a district hospital.

At Zx health facility, one day a week was allocated for review (follow‐up) appointments, but new clients could be registered and obtain ART on the same day. Challenges during 2017 included sporadic low stocks of anti‐retroviral drugs (ARVs); in August 2017, clients were rationed to a two‐week drug supply. Power cuts undermined data entry and laboratory tests. Laboratory Staff members said they sometimes experienced a shortage of reagents. Congestion and long waiting times, especially at registration, and difficulties retrieving client files were also observed. PLHIV arrived from 6.00 am onwards and by the time the clinician started seeing clients at approximately 9.30 am, the ART clinic was crowded with about 50 to 80 clients. Any PLHIV who had missed a prior appointment was attended to last. The pharmacist gave transient PLHIV from outside Zx who had run out of ART a two‐week drug supply. Health staff members said that factors that had helped with clinic congestion included the re‐opening of the government facility near the community boundary, conducting CD4 tests every day, registering PLHIV daily and the assistance of community lay workers (CLWs).

### 3.2 Description of case‐study participants

All but one of the participants were middle‐aged (36 to 44 years). One was a 21‐year‐old woman. Four of the six participants were heads of the household (see Table 1). Two participants owned their plots of land, three of them lived on family plots and one rented. None of them lived in concrete brick houses or had water or electricity in their house. They used near‐by communal water points, candles for light and charcoal for cooking. Three had their own pit latrine, the others utilized communal pit latrines. The smallest household had four members and the largest thirteen. Two participants were married, two widowed, another divorced and the other effectively separated. Being a single parent was experienced as a “struggle” by three participants. All the women spoke about being physically abused by their husbands, and two of the men were open about “beating up” their wives when drunk. Three participants (two men, one woman) regularly drank alcohol, and one struggled with binge drinking. Many of the children were under the age of 18, three households had adult children aged 19 to 21 of whom two contributed to household expenses. Three households had at least one child of school age who they could not afford to send to school. Only one participant could support higher education funding requests. Three households were noticeably food insecure, usually having two meals a day. All households got assistance from neighbours, church members and other relatives, sometimes reciprocating this assistance when they could.

**Table 1:** Demographic profile and livelihood mobility of the six participants (names are pseudonyms)

Participant				HH composition	HH location (estimate)	Livelihood mobility
Name	Age	ART status	HH role			
Ronny (Male)	36	Defaulter Restarted ART	HH head Married	6 HH members: participant, wife, 4 children (ages: 15, 12, 7, 7); 2 in primary school but repeated grades; 2 of school age but not yet enrolled	Main road – 1.2 km Health facility – 11 km NGO ART centre – 1 km Water points – 100 m Market – 12 km	Urban‐urban and urban rural Commuted to work in town as tailor + seasonally migrated to trade/barter maize for clothes in rural farms during harvest (four months). Casual work in rural mine during rains (three months). Wife helped with tailoring in town & rural area
Molly (Female)	44	Defaulter	HH head Widowed	4 HH members: participant, 3 children (ages: 19, 17, 10), eldest stopped school in grade 9, others at secondary and primary schools. HH head's husband died in 2006. 19‐year‐old son moved out for a short time	Main road – 1 km Health facility – 1 km NGO ART centre – 12 km Water point – 500 m Market – 1 km	Within local urban area. Moved about selling assorted fruits within community and in town; travelled outside town to attend church meetings and funerals. Son gave sporadic support & started to pool capital with mother to buy and sell fruit
Denny (Male)	38	ART	Dependent Widowed	8 HH members: participant, brother (HH head), brother's wife and 5 children (ages: 16, 13, 8, 5, 1) – 2 in primary school, 1 of school age but not enrolled. Wife's mother & sister lived on plot. Participant's wife died in 2013, he has 2 children (ages: 6, 9) in village with wife's mother	Main road – 1.2 km Health facility – 1.2 km NGO ART centre – 11 km Water point – 10 m Market – 3 km	Within local urban area and urban‐rural Security guard in Lusaka until very sick. This year moved around within community helping in household & with two market stalls. Brother promised start‐up capital to assist with trading in maize. In June moved to rural area to work in cousin's shop
Pretty (Female)	41	ART	HH head Divorced	10 HH members: participant, 5 children (14, 8, 6, 4, 2), young sister and spouse, 1 brother and 1 sister in college. 2 children in school, 1 re‐enrolled in school with help of boyfriend. HH head divorced husband in June 2016	Main road – 1.3 km Health facility – 9 km NGO ART centre ‐ 7 km Water point 500 m Market – 15 km	Urban‐rural. Travelled outside town to border town to trade in fish, and to rural area to buy maize and wild caterpillars. In last year, travelled away six times for a week to trade. Young sister & husband contributed to household
Simba (Male)	44	Non‐linker	HH head Married	11 HH members: participant, wife, father, 8 children (19, 16, 13, 9, 6, 4, 3, 3mths), 4 children in school	On main road Health facility – 12 km NGO ART – centre 3 km Water point – 25 m Market –15 km	Urban‐rural. Migrated to fishing camp for two to four months at a time and then to fish markets for over two weeks. Rested for one month in‐between at home & during the fish ban (December to March). Fish stocks low, “we used to make good money, now it is quite hard.” Wife traded in fruits, routinely travelled away to buy fruit and returned to sell
Mutinta (Female)	21	Non‐linker	Dependant Married	13 HH members: participant, mother, stepfather (HH head), participant's 2 daughters (ages: 4, 2), 5 stepsisters (ages: 15, 7, 6, 3, 4), stepfather's nephew (young adult), stepfather's 2 nieces in school. Children in school. Participant's husband absent since May 2016; in jail for assault since November 2016. Never lived together	Main road – 3 km Health facility – 4 km NGO ART centre – 13 km Water point 25 m Market – 2 km	Within local urban area and urban‐urban. Moved about selling groundnuts within community and in town centre. In town from 7 am to 5 pm when selling. Travelled outside town to ask for money from relatives, met boyfriends in town & at their homes

All six participants were long‐term residents of Zx, with strong ties to rural areas (where they were brought up and/or traded) and two also spent long periods working in Lusaka. They all had strong local social networks. Relationships with spouses or sexual partners were often tenuous for women, with gender‐based violence and HIV heightening these tensions.

The participants and their households reflected the typical circumstances of many Zambians. They were reliant on the informal economy 17 and they were close to or beneath the poverty line (based on location, food expenditure, average meals a day, access to water, toilets and energy, housing type) 18. Despite the stability given to them by families and long‐term residence and some improvements in road and energy infrastructure, their poverty (coupled with rising costs of living) meant that their lives were precarious, and they were used to relying on their own efforts to survive.

### 3.3 The spinning plates

Household responsibilities, livelihood mobility and health/HIV experiences converged and were experienced daily alongside each other (see Tables 1, 2, 3).

**Table 2:** Health and HIV profiles of six participants

Participant	Health of participant	History of HIV testing	Contact and/or contemplation of ART services
Ronny	2013 very sick, “felt like a paper weight,” “no balance,” “body weakness.” Moved to Zx from Lusaka. Felt much better after starting ART (2014). After defaulting, experienced constipation, malaria, headache, loss of appetite and weight. One week into re‐starting ART said he was doing well	HIV Testing: Disbelief when tested HIV negative twice before testing HIV positive with HPTN071 community lay workers (HPTN071 CLWs). (2014). “I was so sick I never thought I could be HIV negative.” Discordant with wife. Children all tested HIV negative. Ronny was the only one LHIV in his household. Wife continuously retested herself & found it hard to believe she is HIV negative	Started, stopped, started ART. Livelihood mobility and household responsibility influenced this pattern. Started ART: December 2014, started ART within a week although CD4 “ok” Stopped ART: In 2016 ran out of drugs while out of town for petty mining and farmed for over three months. Took one week's worth of drugs with him but away for longer than intended. Took remaining drugs when returned home. Lost ART card. Reluctant to restart for fear of being reprimanded by HCW who “look down on him” and “reprimand so much” HPTN071 (CLWs). encouraged him to find card and restart. Advocated taking ART, but also says that some PLHIV “would rather die than live like this taking ART.” Claimed that some PLHIV take ART with alcohol but “not me” Restarted ART: April 2017, restarted ART from NGO health facility located much closer to his home. Ronny said he was also avoiding the queues at the main clinic. August 2017, in rural area with no ART services but planned to cycle back to collect ART once his 2‐week supply runs out, leaving wife there to run business
Molly	Frequently fell ill in 2015. Suffered from muscle pains, headache, stomach aches and sores on her “private parts.” Felt better once she started ART. In October 2016 developed chest pains and coughed up blood. Diagnosed with TB. Struggled with side‐effects of medications and stopped all medication. She said, “now I only feel pain in my heart” (emotional pain). In February, she “feels well” but by August 2017, she complained of fatigue, body pains & nightmares which she managed with sleep, warm water, massage and prayer	HIV testing: Husband HIV positive and died in 2006. Tested HIV positive at ante‐natal in 2007 when pregnant. 10‐year‐old daughter tested HIV positive with HPTN071 (CLWs) & was informed of her status by child counsellor at clinic	Started, stopped ART. Household responsibility influenced this pattern Starting ART: After falling sick in 2015, told to start “regardless of CD4.” Said that “all the pain reduced” and infection in her “private parts” disappeared when she was on ART. In November 2016 diagnosed with TB (“coughed out blood”) and put on TB medication which made her “black out and weak.” CLWs told her to continue taking drugs and humiliated her (see main text) Stopping ART: Decided to stop taking both TB and ART after being humiliated. “Bad reception made me stop taking ART,” she repeatedly stated. Initially she seemed to consider re‐starting ART if she fell sick again, but is also put off by the long queues, side‐effects, and needing to make a living. “I need to use that waiting time to sell to fend for my children,” Molly explained. Her 10‐year‐old daughter did not start ART either despite the advice of health workers. Molly does not want her to start at Zx health facility because of the “bad reception.” The daughter said she will start when she wants to, “maybe” once she reaches 12 years
Denny	Ill in 2015 with stomach pains and STI; treated with traditional medicine. Fell extremely sick late 2016 with “stomach pains” and lost weight and strength. Regained strength and stomach pains go away on ART. In April 2017 suffered from malaria and is weaker. By May 2017 his health seemed restored although food is short in the household. Participant disclosed to his younger and older brothers, to a close friend, and to his sister and mother	HIV testing: Tested HIV negative early in 2017 while very sick in Lusaka. After moving to his brother's house in Zx, his brother intercepted HPTN071 (CLWs). on the road and asked them to test Denny. This time he tested HIV positive. His brother and family repeatedly tested with HPTN071 (CLWs). and his young sister is LHIV	Started ART. Livelihood mobility and household responsibility influenced decision to move away from easier access to ART, making him more vulnerable to stopping ART Starting ART: After four days of being diagnosed with HIV, he started ART at Zx health facility. Recalled skipping one dose which he forgot to take when watching football and had to wait until the next day to take ART at a prescribed time. Advocated taking ART early because “delaying makes the condition worse” Considered consuming too much alcohol and being on ART “a problem,” although was told he could drink alcohol and take ART as long as he did not get drunk After three months on ART, he moved away to a rural area to work. According to family, there were no ART services where he had moved to and his brothers and cousin collected ART for him from Zx health facility and took it to him
Pretty	Before she started ART she used to feel weak and said she was “not well” when she tested for HIV. In 2017, she felt like she has “much energy.” When she found out she had HIV, she was “very sad” but said the HPTN071 (CLWs) really helped her “to accept.” She talked about HIV with two friends who are also LHIV. Participant disclosed to her ex‐husband, young sister, her best‐friend and another two friends LHIV, her boyfriend, a young sick widow and everyone in her household. The latter reminded her to take her drugs		Started ART. Adjusted livelihood mobility to ART access. Starting ART: Since 2014 when pregnant had taken ART “consistently” from another health facility which was closer to where she lived. She always collected her ART herself and had her “vitals” done. When she travelled away for trading, she limited her time away (from one to three weeks), moved with all her drugs (“I do not carry and leave some. No!”) and disclosed to a colleague she travelled with just in case of health complications. Complained of long queues at the health facility and said the “professional” healthcare workers were “harsh” but said she “always makes time” to collect her drugs
Simba	Suffered from TB in the past. Said he “feels strong” but on second visit complains of headache, stomach ache and coughing. Wife also not well at second visit; she looked frailer. Participant disclosed to his wife, and friend/colleague fisherman	HIV testing: Tested by HPTN071 (CLWs). at home with wife in 2014; both tested HIV positive	Not started ART. Livelihood mobility and household responsibility influenced this decision Referred twice by HPTN071 (CLWs) to Zx health facility. Told by HPTN071 (CLWs). that he should “go through investigations if I do not want to start ART.” Went once but did not find HPTN071 (CLWs) so came home. Had not yet gone back because “still feeling strong” and prompted to start ART “only if sick often.” Heard about “immediate ART” recently and believed that “drugs work” and that “health workers are fine.” No ART services in swamp (where he fished) although “a lot of people on ART there.” Wife said she will start ART when husband starts or when he gets sick. They would go to a health facility closer to their home if they started ART
Mutinta	Complained of stomach aches, painful feet and pain under the breasts. Active and looked well. Mutinta disclosed to her mother, mother‐in‐law and close childhood friend	HIV testing: Tested at antenatal and family planning clinics from 2012 onwards. In August 2016, HPTN071 (CLWs). tested almost everyone in her household and Mutinta tested positive. Did not know the HIV status of anyone else in the household. Re‐tested elsewhere outside Zx after two months to re‐confirm	Not started ART. Deference to position in the household and livelihood mobility influenced this decision Put on Septrin (cotrimoxazole) for three months. Her mother‐in‐law wanted her to wait for her husband to come out of prison and re‐test with him, so they can start ART together. Felt this was “unfair.” She stated there is “nothing to fear” about taking ART, that if you “wait to get sick you risk dying” but “when you start there is no stopping. Once you stop taking the drugs this gives problems.” Said that she would “go on Monday” to another health facility but never went during period of visiting her. Occupied with trading and long queues at ART clinic took time away from livelihood activities. She was also concerned of involuntary disclosure due to location of ART clinic. She said she might start ART when spouse gives permission when he gets released from jail

**Table 3:** Spinning plates

Name	Household responsibility and daily routine type	Livelihood mobility	HIV management	Balance/topple
Ronny	Mostly **flexible daily routine**,** sometimes chaotic.** Extensive social networks & binge drinker, 4 younger children, renting house, mostly food secure, married, relationship with HIV negative ‐ wife up and down, had other sexual partners. Sometimes he & his wife absent at the same time	Mainly tailoring & bartering skills and although travelled away for up to four months, mostly predictable mobility to regularly visited places until took on casual work in mines which was chaotic	Has experienced extreme illness, started, stopped & started ART. Wife & HPTN071 (CLWs). prop up his health. Limited time to access ART; collecting drugs by biking from rural area to Zx proved challenging	Recent past saw topple in livelihood mobility & then Health/HIV. By August 2017, a delicate balance between all three
Molly	Mostly **rigid daily routine.** More limited social network but strong church membership, 2 older & one younger child (latter LHIV), family plot, housing poor, food insecure, widowed, no sexual partners, elder son contributed to household, elder daughter gave emotional support	Initially selling ripe fruit locally & with small returns & capital. Son's support for her business could improve returns. Her role was to sell	Health improved on ART but bad experience of side‐effects of TB medication & in Zx health facility pushed her away from bio‐medicine (and off ART) towards faith‐healing and self‐management. Frail health by August 2017 & selling exhausted her	Health/HIV vulnerable to toppling in August 2017 by not resuming ART. Although poor, other “plates” propped up by church and adult children
Denny	Mostly **flexible routine at first** which **became more chaotic** when shifted household & location again. More limited social network as moved back to area & adult dependent, brothers were very supportive but brother also struggled financially, family plot, food insecure, widowed, no sexual partners, could not afford to have his children live with him	Tried to learn new livelihood options & assisted where he could with help of cousin & brothers. Moved to rural area to work in cousin's shop. Not independent so vulnerable to being moved around	Had experienced extreme illness but once on ART regained strength. However, reliance on his close family to collect ART and bring it to him deepened his dependency on others. Drinking and smoking habits and low self‐esteem threatened his ART adherence	Past few years have seen all three plates topple and fall. Propped up by close family & a hard working personality, but any one plate vulnerable to toppling
Pretty	Mostly **flexible routine.** Extensive social network, 5 younger children, owns plot & reasonable house, food secure, divorced, boyfriend LHIV, responsible for siblings in college, adult sister & husband stayed with her & contributed to household. Shift to boyfriend's house more unpredictable	Routine & relatively predictable travel every couple of months for one to three weeks for trading in fish and/or maize. Travelled with woman companion	Health much better since started ART in 2014 and very pragmatic and organized with her management of HIV. Portrayed a sense of therapeutic citizenship. Sensitive to what her boyfriend thinks of her HIV status	Until August 2017, she had good balance across plates. However, shift to her boyfriend's house could make her household responsibility a bit more unstable, but plates still felt steady and balanced over all
Simba	**Flexible daily routine becoming more chaotic.** Extensive social network, one adult & 7 younger children, owned plots, married, HIV positive wife very supportive, food insecure. He & his wife often absent at the same time. Had some land assets & a resourceful personality	Routine and relatively predictable extended periods away in fishing camps (up to nine months) & travelled far to sell fish. Fish stocks low. Wife also routinely travelled to buy fruits to sell locally	Not yet linked to ART & time was very compressed to access ART. No ART services in fishing camp. Health was getting frailer, and a fishing accident in 2015 was another setback for his health. Wife also not well and not on ART	Health/HIV plate felt the most likely to topple, and this would make the livelihood mobility very challenging and his household responsibilities were extensive. Livelihood mobility plate also more off balance with low fish stocks leading to longer periods in the camps
Mutinta	More **chaotic daily routine**. Extensive social network but dependent and young with small children and living in a large household, mother and mother‐in‐law supportive, mother's household food secure, sexual partners	Little control over livelihood and accompanying mobility, took up opportunities where she can (groundnuts, sexual exchange, asking relatives for money)	Well‐informed but not yet started ART, partly because her mother‐in‐law asked her to wait for her estranged husband to get out of prison. Health seemed stable. Did not use condoms in sexual relationships or inform partners of her status	Her future seemed quite unpredictable, and her household and livelihood plates the most likely to topple

### 3.4 Household responsibility

All six participants carried household responsibilities: four were household heads and accountable for food security, water and firewood, school fees for children and providing shelter. The two participants (Denny and Mutinta) who were not household heads had two children each to support and contributed to their household's daily needs.

Denny was a 38‐year‐old widower whose wife died of HIV in 2013. Although he had also been sick due to HIV, he started ART in 2016 within a week of being diagnosed and regained health. He had two children who were living with his mother in‐law. At the time of the first visit, he was living with his elder brother's family in a two‐room house. Due to limited employment opportunities in Zx, he repeatedly said he would travel out of town as soon as he fully recovered to search for employment so that he could meet his needs as well as those of his two children. Although he was not living with his children, his mother‐in‐law required him to support his children (providing food, clothes and school fees). By the third visit, Denny had travelled to a farm in a rural area near Zx to work in a cousin's shop and hoped to also do some farming piecework. There were no ART services in this rural area, and he was dependent on family members collecting ART from the health facility in Zx and bringing to him. Reflecting on his dependency on others, he said that regaining health would allow him to pick up his responsibilities as a father and move elsewhere to earn a living and find his own shelter.

Five of the six participants had made providing for their households and their dependents a priority over their own health, taking decisions to delay, miss or stop taking treatment to make ends meet for their households. Molly, for example was a 44‐year‐old widow living with HIV who had three teenage children. Her youngest child was perinatally infected with HIV. The oldest child was forced to stop school due to financial constraints and started travelling to Zambia's capital to buy fruit which Molly resold by walking around the community and in the town centre. In 2015, after frequently falling sick and being diagnosed with HIV, Molly started ART. In 2017, she was diagnosed with tuberculosis (TB) but taking drugs for both HIV and TB made her feel very sick and she could not walk around to sell her commodities. A decision to stop taking TB and HIV drugs was influenced by these factors coupled with being fed up with long queues at the health facility and a stigmatizing incident there. “I need to use that waiting time to sell to fend for my children,” she explained. She also felt she needed to increase her income to improve her diet to take ART:

I will start ART again, first [,] I want my business to stabilize because business has been hard for me of late…Besides I need to work to earn money so that I will be able to buy food such that as I take the medication I do not get sick, it is not okay to take strong drugs without good food or poor nutrition.

### 3.5 Livelihood mobility

The main reason for mobility was to raise money for household basic needs (food, education, shelter, water, laundry, soap), as well as for capital needed for business ventures, recreational pursuits and social obligations. For two participants, poor health was a major motivation for moving from Lusaka back to family in Zx to be looked after. Their decisions around HIV (including starting treatment and routinely accessing drugs) were directly affected by the livelihood demands that involved urban‐urban and urban‐rural mobility.

One routine urban‐urban mobility pattern was moving within Zx to the market and around the community and outside Zx to a nearby town centre. Molly and Mutinta used “small capital” (US$10‐15) for mobile businesses which entailed buying groundnuts or fruit for resale door‐to‐door, in markets and town centre streets, selling from 7 am to 5 pm during the week. Small profit margins, long days and geographical distances (into town and back and around the community) undermined their motivation to take ART. For example, Mutinta was occupied with trading and increasingly used transactional sex in the town centre to survive and said that the long queues at the health facility took time away from her business which was “not doing well.”

Bigger returns were made by travelling further afield to urban and rural areas demonstrating urban‐rural mobility patterns. Three participants routinely left Zx to make a living. Ronny was a tailor working within Zx, in the town centre and in a rural area, shifting to a temporary shelter in a rural area around the harvest season for four months to barter making or mending clothes for maize grain, which he would then resell in Zx. During the rains, he had also taken on three months casual work in a rural mine. Pretty travelled for a week at a time roughly every two months either to national border posts or to rural areas to trade in dried fish and maize. She supplemented her household income with contributions from her younger sister and fisherman brother‐in‐law (who stayed with her).

Fishing was the main occupation of Simba. He spent about nine months a year fishing in the swamps and travelling to border posts and main towns to sell fish and this affected his access to HIV services since there was nowhere to access ART in the fishing camps. His wife, who was also LHIV and not on treatment, travelled to buy fruit from a distant town to resell in Zx, and was often away for two weeks a month. Simba explained that they will start ART with each other when one of them falls sick. In the meantime, he needed to prioritize household needs:

If I start now, where I go it is far and there is no ART centre. What happens when I run out of drugs? It is better I use my body to feed my family when I am still feeling strong.

Simba's predicament was echoed in Ronny's experience of stopping treatment for a period of six months because he ran out of drugs when he travelled from Zx to work in an informal copper mine. On his return to Zx, he further delayed resuming ART because he worried that health workers would reprimand him. Eventually he decided to access ART from the local NGO. He narrated:

I lost my ART identification number, and where I went there are no ART services, and I fear going to a public health ART centre because they would tell me off for treatment disruption.

### 3.6 Health and HIV

It was usually frequent illnesses, and for two participants, extreme illness, that prompted a decision to test for HIV (see Table 2). Most had found out they were LHIV within the last three years by testing at home with HPTN071 Staff members. The exception is Molly who found out in 2007 through ante‐natal care services. Simba, Denny and Molly have other family members LHIV that they stay with (a wife, an adult sister and a 10‐year‐old daughter respectively). All participants had disclosed to at least one other person in the household.

Although not all were on ART, all believed in ART, advocating taking it early and “consistently.” ART was acknowledged as improving health and pushing illness away. Of the three participants not on treatment by August 2017, two (and one of their spouses) were not well. Denny had started on treatment recently when extremely ill and was frail, although slowly regained strength.

Worries about being seen accessing ART at Zx health facility were experienced either as a personal apprehension of participants or as something others worried about. Opting for “quieter” health facilities outside the Zx boundary with shorter queues was one strategy to avoid “being seen.” Simba recalled PLHIV being made fun of for taking ART, and Molly said she was ridiculed by volunteers at Zx health facility when she fell sick with TB while on ART. She recollected, “they said that even the air they breathe has been polluted due to the high viral load I have…they said I have infected a lot of people.”

Experience with the HIV services at Zx health facility were chequered. Participants’ complaints included long queues, rigid opening hours and/or health workers starting work late and blood results being lost. Although one participant said the healthcare workers were “fine,” three others recalled being reprimanded and treated “harshly.” One of the main reasons for being reprimanded was missing review appointments. However, Ronny's wife understood the frustrations of health workers faced with clients who do not come for their review appointment. She commented, “I think when they reprimand the patients it is to encourage them about their health, to look after your family.”

After the difficult experience at Zx health facility, Molly relied on faith‐healing and good nutrition which she found “more convenient” and “flexible.” She explained how she can now “sell” from Monday to Friday, and still attend evening prayers: “When I attend church, I am even able to do my business because time is flexible than when I have to go and queue up at the clinic eating into my business time.” At church on Sunday, she “benefits” not only from faith but also from testimonies on faith‐healing and a counselling group for PLHIV.

Pretty, who travelled regularly but for limited periods (one to three weeks), travelled with all her drugs and with a woman trader companion who knew her HIV status. She “always makes time” to collect her drugs, choosing to access them from another health facility which was closer to her home. Explaining how she managed to stay on treatment both at home and when travelling away from home, she said:

My children remind me to take medicine when I forget, and when I am travelling or away to sell my commodities my friend reminds me because I disclosed to her. She is also HIV positive but not on ART. I also make sure I collect enough drugs for the period I am away.

When Denny shifted to work in a rural area with no ART services, his brother and cousin said they would collect his drugs and take them to him. But, this arrangement did not always work. He lamented that:

My cousin stopped going to town frequently, missed on collection of my drugs and my brother was out of town for a long time so my treatment was disrupted.

Ronny, faced with the same issue of residing (temporarily) in a rural area, resolved to cycle from the rural area to collect his drugs, leaving his wife to manage the business when he was away.

## 4 Discussion

The case‐studies of six Zambian PLHIV demonstrate how time to access ART was compressed by household responsibilities and the need to earn a living moving around, often away from home. At the health facility, lengthy waiting times, routine appointments, stigma linked to health facility space and health worker attitudes affected access. Using the typology of rigid, flexible and chaotic routines 9 and the spinning plate analogy shows how each participant could not afford to focus on health and HIV exclusively (see Table 3). Each “plate” of household responsibility, livelihood mobility and HIV‐management needed to be in balance; the tendency was to focus on the “plate” most vulnerable to toppling at the expense of the others. More chaotic and varied routines limit the time to access ART and rigid routines can be both oppressive and/or facilitate ART 9.

Intervening to support household responsibility and livelihood options is challenging. There is no comprehensive welfare support system in place in Zambia, although the government is planning to introduce a social cash transfer scheme 34 which aims to provide small bi‐monthly cash payments to households experiencing severe poverty. We argue in this context that health service delivery needs adjustment to allow ART adherence, since livelihood mobility is an unalterable “temporal and spatial reality” [24 p862]. One area of intervention therefore could be in alternative health delivery methods. Based on the time‐geography of PLHIV in California, Takashashi et al. [9 p862] suggest that service providers “make efforts to explore the daily routines of …and restructure their organizational strategies and practices to take account of these temporal and spatial realities.” As Taylor et al. [24 p293] state, based on patterns of mobility among Dominicans LHIV and on ART in New York City, “finding novel approaches to combat mobility‐induced barriers to care, will be critical to the success of treatment programmes for HIV.”

Leading precarious and mobile daily lives is a poor fit with the structure of HIV services 2, 9, 11 that require strict adherence and predictable routines, creating challenges both for PLHIV and health workers responsible for ART delivery. In Zambia, there are HIV delivery initiatives allowing travelling PLHIV (and family members) 35 to collect extra supplies of ART and access ART from a local NGO or outside the community boundary. However, the health worker practice of reprimand 20, 36, 37 towards those who miss review appointments, also noted by others in Zambia 37, 38 and Kenya 11, intermittent drug stock shortage (and resulting rationed supply of drugs to clients), limiting ART provision to particular days and the lack of ART services in rural areas 3, 39, 40 and fishing camps indicate areas that require practical space and time strategies.

### 4.1 Limitations

The data are limited by the small number of participants. However, the granular analysis and case‐study approach provide detail on prevalent mobility patterns. A longitudinal approach, extending the period of fieldwork, would have illustrated the spinning plate analogy more comprehensively.

## 5 Conclusions

Given the inescapable role of livelihood mobility in the lives of many Zambians LHIV, the most promising interventions for sustained ART access and health are more client‐centred care through a combination of changes that allow for mobility and flexibility and greater understanding between health workers and clients. ART services need to be in closer proximity to where livelihood activities take place 40 and open in the evenings and at weekends. Providing clients with a map of where they can access ART if they run out of drugs when travelling, giving more long‐term supplies of ART, an effective centralized client health information system and not restricting ART provision to a particular week day could be other practical strategies to address the needs of PLHIV on ART 41, 42. Health services need to adjust and adapt programming and service delivery to the “every day geographies” [9 p861] of many people living with HIV (PLHIV) in Zambia and their different degrees of inescapable routine mobility.

## Competing interests

There are no conflicts of interest for any authors.

## Authors’ contributions

FN and VB were both involved in the design of the qualitative cohort data collection process, FN collected the data in this analysis, VB and FN analysed the data together and VB led on the drafting and analysis frame of this manuscript with close support from FN. AT assisted with the literature review and revised and reviewed manuscript drafts. MS was involved in the qualitative cohort data collection process, conducted and wrote up earlier data on the same community and reviewed the content of this manuscript. GH led in the design of the qualitative cohort and supported the analysis and content of this manuscript. JS gave technical support to the qualitative cohort at all stages and supported the analysis approach and editing for this manuscript. HA, RH and SF designed the HPTN071 intervention and have oversight of all research activities.

## Funding

HPTN 071 is sponsored by the National Institute of Allergy and Infectious Diseases (NIAID) under Cooperative Agreements UM1‐AI068619, UM1‐AI068617, and UM1‐AI068613, with funding from the U.S. President's Emergency Plan for AIDS Relief (PEPFAR). Additional funding is provided by the International Initiative for Impact Evaluation (3ie) with support from the Bill & Melinda Gates Foundation, as well as by NIAID, the National Institute on Drug Abuse (NIDA) and the National Institute of Mental Health (NIMH), all part of NIH. The content is solely the responsibility of the authors and does not necessarily represent the official views of the NIAID, NIMH, NIDA, PEPFAR, 3ie, or the Bill & Melinda Gates Foundation.

## References

[1] UNAIDS . Zambia Country Overview. UNAIDS: Geneva; 2018 Available from: http://www.unaids.org/en/regionscountries/countries/zambia

[2] Tanser F , Bärnighausen T , Vandormael A , Dobra A . HIV treatment cascade in migrants and mobile populations. Curr Opin HIV AIDS. 2015;10(6):430 Available from: http://content.wkhealth.com/linkback/openurl?sid=WKPTLP:landingpagean=01222929-201511000-00007 26352396 10.1097/COH.0000000000000192

[3] Seeley JA , Allison EH . HIV/AIDS in fishing communities: challenges to delivering antiretroviral therapy to vulnerable groups. AIDS Care. 2005;17(6):688–97. Available from: http://www.tandfonline.com/doi/abs/10.1080/09540120412331336698 16036255 10.1080/09540120412331336698

[4] De Bruijn M , van Dijk RA , Foeken DE . Mobile Africa: changing patterns of movement in Africa and beyond. Boston: Brill; 2001.

[5] Kagee A , Remien RH , Berkman A , Hoffman S , Campos L , Swartz L . Structural barriers to ART adherence in Southern Africa: challenges and potential ways forward. Glob Public Health. 2011;6(1):83–97. Available from: http://www.tandfonline.com/doi/abs/10.1080/17441691003796387 20509066 10.1080/17441691003796387 PMC3056922

[6] Lehane E , McCarthy G . Intentional and unintentional medication non‐adherence: a comprehensive framework for clinical research and practice? A discussion paper Int J Nurs Stud. 2007;44(8):1468–77. Available from: http://www.ncbi.nlm.nih.gov/pubmed/16973166 16973166 10.1016/j.ijnurstu.2006.07.010

[7] Blalock AC , McDaniel JS , Farber EW . Effect of employment on quality of life and psychological functioning in patients with HIV/AIDS. Psychosomatics. 2002;43(5):400–4. Available from: http://linkinghub.elsevier.com/retrieve/pii/S003331820270368X 12297609 10.1176/appi.psy.43.5.400

[8] Weiser S , Wolfe W , Bangsberg D , Thior I , Gilbert P , Makhema J , et al. Barriers to antiretroviral adherence for patients living with HIV infection and AIDS in Botswana. JAIDS J Acquir Immune Defic Syndr. 2003;34(3):281–8. Available from: http://content.wkhealth.com/linkback/openurl?sid=WKPTLP:landingpage&an=00126334-200311010-00004 14600572 10.1097/00126334-200311010-00004

[9] Takahashi LM , Wiebe D , Rodriguez R . Navigating the time‐space context of HIV and AIDS: daily routines and access to care. Soc Sci Med. 2001;53(7):845–63. Available from:http://www.sciencedirect.com/science/article/pii/S0277953600003634 11522133 10.1016/s0277-9536(00)00363-4

[10] Harvey D . The condition of postmodernity: an enquiry into the origins of cultural change. Cambridge, MA: Blackwell; 1990.

[11] Prince R . HIV and the moral economy of survival in an East African city. Med Anthropol Q. 2012;26(4):534–56. Available from: https://doi.org/doi.wiley.com/10.1111/maq.12006 23361884 10.1111/maq.12006

[12] Kalofonos I . “All I Eat Is ARVs!” The paradox of AIDS treatment interventions in Mozambique. Med Anthropol Q. 2010;24(3):363–80. Available from: http://onlinelibrary.wiley.com/doi/10.1111/j.1548-1387.2010.01109.x/abstract 20949841 10.1111/j.1548-1387.2010.01109.x

[13] Nguyen VK . The republic of therapy: triage and sovereignty in West Africa's time of AIDS. Duke University Press: North Carolina, United States; 2011 Available from: http://www.tandfonline.com/doi/abs/10.1080/1744169220 11.594075

[14] Castro A . Adherence to antiretroviral therapy: merging the clinical and social course of AIDS. PLoS Med. 2005;2(12):1217–21. 10.1371/journal.pmed.0020338 PMC1240055 16187735

[15] Mizuno Y , Purcell D , Borkowski TM , Knight K ; SUDIS Team . The life priorities of HIV‐seropositive injection drug users: findings from a community‐based sample. AIDS Behav. 2003;4:395–403. Available from: http://link.springer.com/10.1023/B:AIBE.0000004731.94734.77 10.1023/b:aibe.0000004731.94734.77 14707536

[16] Fassin D . Another politics of life is possible In: Fassin D, editor. Theory, Culture & Society. Oxford: Wiley; 2009 p. 44–60. Available from: http://journals.sagepub.com/doi/10.1177/0263276409106349

[17] Mills EJ , Nachega JB , Bangsberg DR , Singh S , Rachlis B , Wu P , et al. Adherence to HAART: a systematic review of developed and developing nation patient‐reported barriers and facilitators. PLoS Med. 2006;3(11):2039–64. 10.1371/journal.pmed.0030438 PMC1637123 17121449

[18] Ferguson J . Expectations of modernity: myths and meanings of urban life on the Zambian Copperbelt (review). Anthropol Q. 2001;74(2):89–90. Available from: http://muse.jhu.edu/content/crossref/journals/anthropological_quarterly/v074/74.2piot.pdf

[19] Hardon A , Dilger H . Global AIDS medicines in east African health institutions. Med Anthropol. 2011;30(2):136–57. 21400350 10.1080/01459740.2011.552458

[20] Ware NC , Idoko J , Kaaya S , Biraro IA , Wyatt MA , Agbaji O , et al. Explaining adherence success in sub‐Saharan Africa: an ethnographic study. PLoS Med. 2009;6(1):e1000011 Available from: http://dx.plos.org/10.1371/journalpmed.1000011 10.1371/journal.pmed.1000011 PMC2631046 19175285

[21] Taylor BS , Garduño LS , Reyes EV , Valiño R , Rojas R , Donastorg Y , et al. HIV care for geographically mobile populations. Mt Sinai J Med. 2011;78(3):342–51. Available from: https://doi.org/doi.wiley.com/10.1002/msj.20255 21598261 10.1002/msj.20255 PMC3100665

[22] Seeley J , Watts CH , Kippax S , Russell S , Heise L , Whiteside A . Addressing the structural drivers of HIV: a luxury or necessity for programmes? J Int AIDS Soc. 2012; 15(Suppl 1):1–4. 10.7448/IAS.15.3.17397 PMC3499845 22905346

[23] Shanaube K , Schaap A , Floyd S , Phiri M , Griffith S , Chaila J , et al. What works – reaching universal HIV testing. AIDS. 2017;31(11):1555–64. Available from: http://insights.ovid.com/crossref?an=00002030-201707170-00007 28471766 10.1097/QAD.0000000000001514 PMC5491236

[24] Taylor BS , Reyes E , Levine EA , Khan SZ , Garduño LS , Donastorg Y , et al. Patterns of geographic mobility predict barriers to engagement in HIV care and antiretroviral treatment adherence. AIDS Patient Care STDS. 2014;28(6):284–95. Available from: http://online.liebertpub.com/doi/abs/10.1089/apc.2014.0028 24839872 10.1089/apc.2014.0028 PMC4046197

[25] Hayes R , Ayles H , Beyers N , Sabapathy K , Floyd S , Shanaube K , et al. HPTN 071 (PopART): rationale and design of a cluster‐randomised trial of the population impact of an HIV combination prevention intervention including universal testing and treatment ‐ a study protocol for a cluster randomised trial. Trials. 2014;15(1):57 Available from: http://trialsjournal.biomedcentral.com/articles/10.1186/1745-6215-15-57 24524229 10.1186/1745-6215-15-57 PMC3929317

[26] Hayes R , Floyd S , Schaap A , Shanaube K , Bock P , Sabapathy K , et al. A universal testing and treatment intervention to improve HIV control: one‐year results from intervention communities in Zambia in the HPTN 071 (PopART) cluster‐randomised trial. PLoS Med. 2017;14(5):1–22. Available from: https://doi.org/dx.doiorg/10.1371/journal.pmed.1002292 10.1371/journal.pmed.1002292 PMC5412988 28464041

[27] Kielmann K , Cataldo F , Seeley J . Introduction to qualitative research methodology: a training manual. UK: Department for International Development (DfID); 2012.

[28] Bond V , Hoddinott G , Viljoen L , Simuyaba M , Musheke M , Seeley J . Good health and moral responsibility: key concepts underlying the interpretation of treatment as prevention in South Africa and Zambia before rolling out universal HIV testing and treatment. AIDS Patient Care STDS. 2016;30(9):425–34. Available from: http://online.liebertpub.com/doi/10.1089/apc.2016.0114 27610464 10.1089/apc.2016.0114 PMC5035365

[29] Stake RE . The art of case study research. London: Sage; 1995.

[30] Seeley J , Bond V , Hoddinott G , Floyd S , Macleod D , Viljoen L , et al. Understanding time needed to link to care and start ART. Paper Presented at: The 9th International AIDS Society (IAS) conference on HIV Science; 2017 Jul 23–7; Paris.

[31] Central Statistical Office . Living conditions monitoring survey report Lusaka; 2015 Available from: http://www.zamstats.gov.zm/nada/index.php/catalog/59/study-description.

[32] Bond V , Chiti B , Hoddinott G , Reynolds L , Schaap A , Simuyaba M , et al. “The difference that makes a difference”: highlighting the role of variable contexts within an HIV Prevention Community Randomised Trial (HPTN 071/PopART) in 21 study communities in Zambia and South Africa. AIDS Care. 2016;28:99–107. 10.1080/09540121.2016.1178958 27421057

[33] Ware NC , Wyatt MA , Geng EH , Kaaya SF , Agbaji OO , Muyindike WR , et al. Toward an understanding of disengagement from HIV treatment and care in Sub‐Saharan Africa: a qualitative study. PLoS Med. 2013;10(1):e1001369. 23341753 10.1371/journal.pmed.1001369 PMC3541407

[34] Ministry of Community Development M and CH (MCDMCH). Harmonised manual of operations, social cash transfer scheme. Department of Social Welfare: Lusaka, Zambia; 2013.

[35] Renju J , Moshabela M , Mclean E , Ddaaki W , Skovdal M , Odongo F , et al. “Side effects “are” central effects” that challenge retention in HIV treatment programmes in six sub‐Saharan African countries: a multicountry qualitative study. Sex Transm Infect. 2017;93(Supplement 3):1–5. 10.1136/sextrans-2016-052971 PMC5739838 28736390

[36] Campbell C , Scott K , Skovdal M , Madanhire C , Nyamukapa C , Gregson S . A good patient ? How notions of “a good patient” affect patient‐nurse relationships and ART adherence in Zimbabwe BMC Infect Dis. 2015;15:1–11. Available from: 10.1186/s12879-015-1139-x 26424656 PMC4589970

[37] Musheke M , Bond V , Merten S . Individual and contextual factors influencing patient attrition from antiretroviral therapy care in an urban community of Lusaka, Zambia. J Int AIDS Soc. 2012;15(Suppl 1):1–9. 10.7448/IAS.15.3.17366 PMC3499928 22713354

[38] Simuyaba M , Bond V , Hoddinott G , Bwalya C , Ndubani R , Ngwenya F , et al. A qualitative study exploring community stakeholder perceptions and experiences with rolling out universal treatment in HPTN 071 (PopART) study sites in Zambia. Paper Presented at: 8th South Africa AIDS Conference; 2017 Jun 13‐15; Durban.

[39] Mutevedzi PC , Lessells RJ , Heller T , Bärnighausen T , Cooke GS , Newell ML . Scale‐up of a decentralized HIV treatment programme in rural KwaZulu‐Natal, South Africa: does rapid expansion affect patient outcomes? Bull World Health Organ. 2010;88(8):593–600. Available from: http://www.pubmedcentral.nih.gov/articlerender.fcgi?artid=2908968&tool=pmcentrez&rendertype=abstract40 20680124 10.2471/BLT.09.069419 PMC2908968

[40] Palk L , Blower S . Mobility and circular migration in Lesotho: implications for transmission, treatment and control of a severe HIV epidemic. HHS Public Access. 2015;68(5):604–8. Available from: http://www.ncbi.nlm.nih.gov/pmc/articles/PMC4365822/ 10.1097/QAI.0000000000000526 PMC4365822 25763787

[41] Prust ML , Banda CK , Nyirenda R , Chimbwandira F , Kalua T , Jahn A , et al. Multi‐month prescriptions, fast‐track refills, and community ART groups: results from a process evaluation in Malawi on using differentiated models of care to achieve national HIV treatment goals. J Int AIDS Soc. 2017;4(S4):41–50. 10.7448/IAS.20.5.21650 PMC5577715 28770594

[42] Ministry of Health . Assessment of the Health Information System in Zambia. Evaluation. Lusaka, Zambia; 2007.

